# Human Multilineage 3D Spheroids as a Model of Liver Steatosis and Fibrosis

**DOI:** 10.3390/ijms20071629

**Published:** 2019-04-02

**Authors:** Piero Pingitore, Kavitha Sasidharan, Matias Ekstrand, Sebastian Prill, Daniel Lindén, Stefano Romeo

**Affiliations:** 1 Department of Molecular and Clinical Medicine, University of Gothenburg, SE-413 45 Gothenburg, Sweden; piero.pingitore@wlab.gu.se (P.P.); kavitha.sasidharan@wlab.gu.se (K.S.); matias.ekstrand@wlab.gu.se (M.E.); 2 Cardiovascular, Renal and Metabolism, IMED Biotech Unit, AstraZeneca, SE-431 83 Gothenburg, Sweden; sebastian.prill@astrazeneca.com (S.P.); Daniel.Linden@astrazeneca.com (D.L.); 3 Division of Endocrinology, Department of Neuroscience and Physiology, Sahlgrenska Academy, University of Gothenburg, SE-405 30 Gothenburg, Sweden; 4 Clinical Nutrition Unit, Department of Medical and Surgical Sciences, Magna Graecia University, 88100 Catanzaro, Italy; 5 Cardiology Department, Sahlgrenska University Hospital, SE-413 45 Gothenburg, Sweden

**Keywords:** NAFLD, NASH, fibrosis, organoids, PNPLA3, fatty acids, liraglutide, elafibranor, vitamin E, obeticholic acid

## Abstract

Non-alcoholic fatty liver disease (NAFLD) is the most common liver disorder in western countries. Despite the high prevalence of NAFLD, the underlying biology of the disease progression is not clear, and there are no approved drugs to treat non-alcoholic steatohepatitis (NASH), the most advanced form of the disease. Thus, there is an urgent need for developing advanced in vitro human cellular systems to study disease mechanisms and drug responses. We attempted to create an organoid system genetically predisposed to NAFLD and to induce steatosis and fibrosis in it by adding free fatty acids. We used multilineage 3D spheroids composed by hepatocytes (HepG2) and hepatic stellate cells (LX-2) with a physiological ratio (24:1). HepG2 and LX-2 cells are homozygotes for the *PNPLA3* I148M sequence variant, the strongest genetic determinant of NAFLD. We demonstrate that hepatic stellate cells facilitate the compactness of 3D spheroids. Then, we show that the spheroids develop accumulations of fat and collagen upon exposure to free fatty acids. Finally, this accumulation was rescued by incubating spheroids with liraglutide or elafibranor, drugs that are in clinical trials for the treatment of NASH. In conclusion, we have established a simple, easy to handle, in vitro model of genetically induced NAFLD consisting of multilineage 3D spheroids. This tool may be used to understand molecular mechanisms involved in the early stages of fibrogenesis induced by lipid accumulation. Moreover, it may be used to identify new compounds to treat NASH using high-throughput drug screening.

## 1. Introduction

Non-alcoholic fatty liver disease (NAFLD) is the most common liver disorder in western countries, affecting 17–46% of adults, and is becoming the major cause of liver disease and transplantation [1]. NAFLD encompasses a wide spectrum of pathologic conditions, including hepatic steatosis and non-alcoholic steatohepatitis (NASH), which can further progress to fibrosis, cirrhosis and hepatocellular carcinoma [2]. Hallmark of this spectrum is an excessive accumulation of fat (steatosis) exceeding 5% of total liver weight [3,4,5]. Hepatic lipid accumulation is deleterious to the liver, giving rise to both morphological and functional perturbations of liver architecture and function [5,6,7,8]. Despite the high prevalence of NAFLD, the understanding of the biology underlying the disease progression is not clear, and importantly there are no approved drug treatments for NASH, the most severe form of the disease.

To understand molecular mechanisms of NAFLD and to identify new drugs several in vitro models of fatty liver disease based on 2D cell culture and in vivo mouse models have been exploited [9,10]. However, 2D cultures do not represent the complexity of human tissues [11], while in vivo animal models often do not mirror human disease due to interspecies differences [12]. Therefore, new in vitro models mimicking liver complexity and better emulating pathophysiology of NAFLD and NASH are highly needed. This need has led to generation of more complex 3D in vitro models that have generated great attention [13]. For instance, 3D cell cultures represent more closely the heterogeneous cell–cell interactions and offer a more similar micro-environment to the in vivo situation with respect to cell shape, adhesion, behavior, topology, and morphology [14]. A complex interplay exists between hepatocytes and hepatic stellate cells (HSCs) in hepatic fibrogenesis [15]. The activation of HSCs after liver injury leads to production of extracellular matrix (ECM) [15] leading to fibrosis and ultimately cirrhosis. Of note, clinical studies show how severe fibrosis is the best predictor of mortality in patients with NAFLD [16].

The liver lobule is formed by parenchymal cells (hepatocytes) and non-parenchymal cells, such as HSCs. The two more abundant cell types in the liver are hepatocytes performing the majority of liver functions and hepatic stellate cells (HSCs) playing critical roles in liver fibrosis [17,18,19]. In normal liver, HSCs maintain a non-proliferative, quiescent phenotype [20]. However, after liver injury HSCs transdifferentiate and start producing extracellular matrix (ECM) proteins [17,19,21,22]. Among ECM proteins, collagens are the most abundant structural protein in the liver. A disproportionate concentration of collagens results in altered cell phenotypes and architectural distortion ultimately leading to cirrhosis.

The rs738409 in the *patatin like phospholipase domain containing 3* (*PNPLA3*) gene encoding for an isoleucine to methionine substitution at position 148 (*PNPLA3* I148M) of the protein is the strongest genetic variation increasing the risk of NAFLD [23]. Studies in murine models showed that this sequence variation increases lipid biosynthesis [24] and that its downregulation is beneficial against fatty liver disease [25,26]. Moreover, carriers of the *PNPLA3* I148M variant have several specific characteristics including a lower hepatic VLDL secretion [27], lower turnover of retinol in stellate cells [28,29,30,31] and a specific signature of circulating lipids [32]. They also display a diverse response to statins [33], fenofibrate and omega 3 fatty acids [34,35] and present more hepatotoxicity after treatment for leukemia [36]. All these findings suggest that *PNPLA3* I148M associated disease represents a separate entity within NAFLD.

In this study, we developed an in vitro model of human NAFLD with genetic predisposition. This model consisted of 3D multilineage hepatic spheroids composed by human hepatocyte (HepG2) and hepatic stellate cells (LX-2) homozygotes for the *PNPLA3* I148M sequence variant. First, we demonstrate that hepatic stellate cells facilitate the compactness of 3D spheroids, indicating that HSCs play an important role in matrix remodeling. Then, we show that the spheroids accumulated intracellular fat and collagen deposition upon exposure to free fatty acids. Finally, this accumulation was rescued by incubating spheroids with liraglutide or elafibranor, drugs currently evaluated in clinical trials for the treatment of NASH and associated fibrosis.

## 2. Results

### 2.1. Co-Culture of HepG2 and LX-2 Cells Facilitates the Compactness of 3D Spheroids

**Figure 1:**
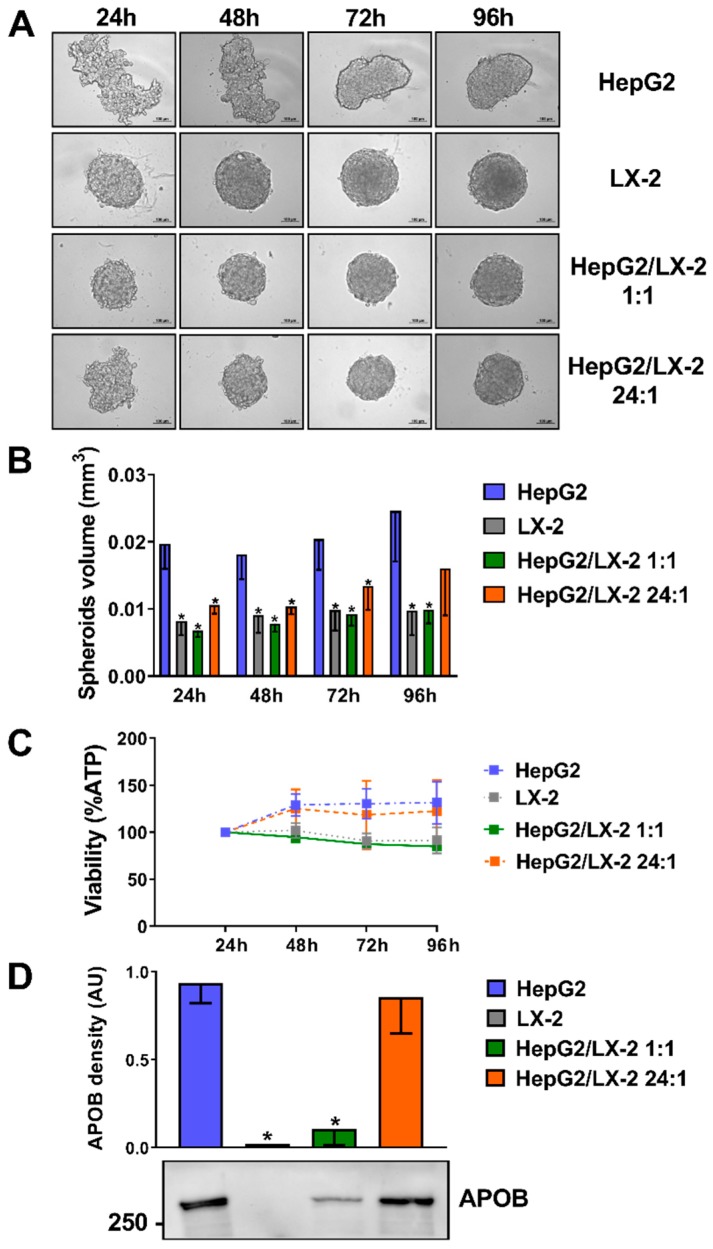
Co-culture of HepG2 and LX-2 cells enhances the compactness of 3D spheroids. (**A**) HepG2 cells, which formed loosely aggregated spheroids alone, were co-cultured as multilineage spheroids with LX-2 cells (forming round-shape spheroids quickly) at a 1:1 and 24:1 ratio for 24, 48, 72 and 96 h. Scale bars in bright-field pictures are 100 μm. (**B**) Spheroid volume was calculated measuring their long and short diameter by ZEN 2.3 Lite software (Zeiss) (*n* = 4). (**C**) Cellular ATP levels normalized to spheroids volumes remained stable throughout 4 days of culture (*n* = 3). (**D**) Apolipoprotein B (APOB) secretion levels in the media fractions measured by Western blotting are proportioned to the percentage of hepatocytes present in the spheroids (*n* = 5). Bars represent mean ± SD. P-values were calculated by Mann-Whitney non-parametric test, (* *p* < 0.05 vs. HepG2). APOB: Apolipoprotein B; AU: arbitrary unit.

To establish the ideal ratio between hepatocyte and hepatic stellate cells in 3D spheroids, we incubated in ultra-low attachment 96-well plates (Corning): (1) human hepatocellular carcinoma (HepG2) cells only; (2) human immortalized hepatic stellate (LX-2) cells only; (3) a combination of HepG2 and LX-2 cells at a 1:1 ratio; (4) a combination of HepG2 and LX-2 cells at a 24:1 ratio. We observed a progressive adhesion of cells forming round-shaped 3D structures up to 96 h of culture. This phenomenon was more pronounced in LX-2 as compared to HepG2 cells. The compactness of the structure of HepG2 cells was profoundly enhanced by co-culture with LX-2 cells even at the lowest ratio of 24:1 (Figure 1A). These data were confirmed by measuring the volume of each spheroid (Figure 1B). To test the cell viability of the spheroids, ATP levels were measured. The ATP levels normalized by volume remained stable (Figure 1C), suggesting no decrease in viability among the different cell compositions of spheroids and time course. Apolipoprotein B (APOB) secretion, as a measure of hepatocyte differentiation and function in spheroids, was measured by Western blotting in the media fractions. The amount of APOB secreted was similar in spheroids composed only of hepatocytes and those with a ratio hepatocytes/hepatic stellate cells 24:1, while it was lower when a 1:1 ratio was used (Figure 1D).

### 2.2. Incubation of Multilineage 3D Spheroids with Fatty Acids Results in Higher Intra-Spheroidal Fat Content and Higher Secretion of APOB

**Figure 2:**
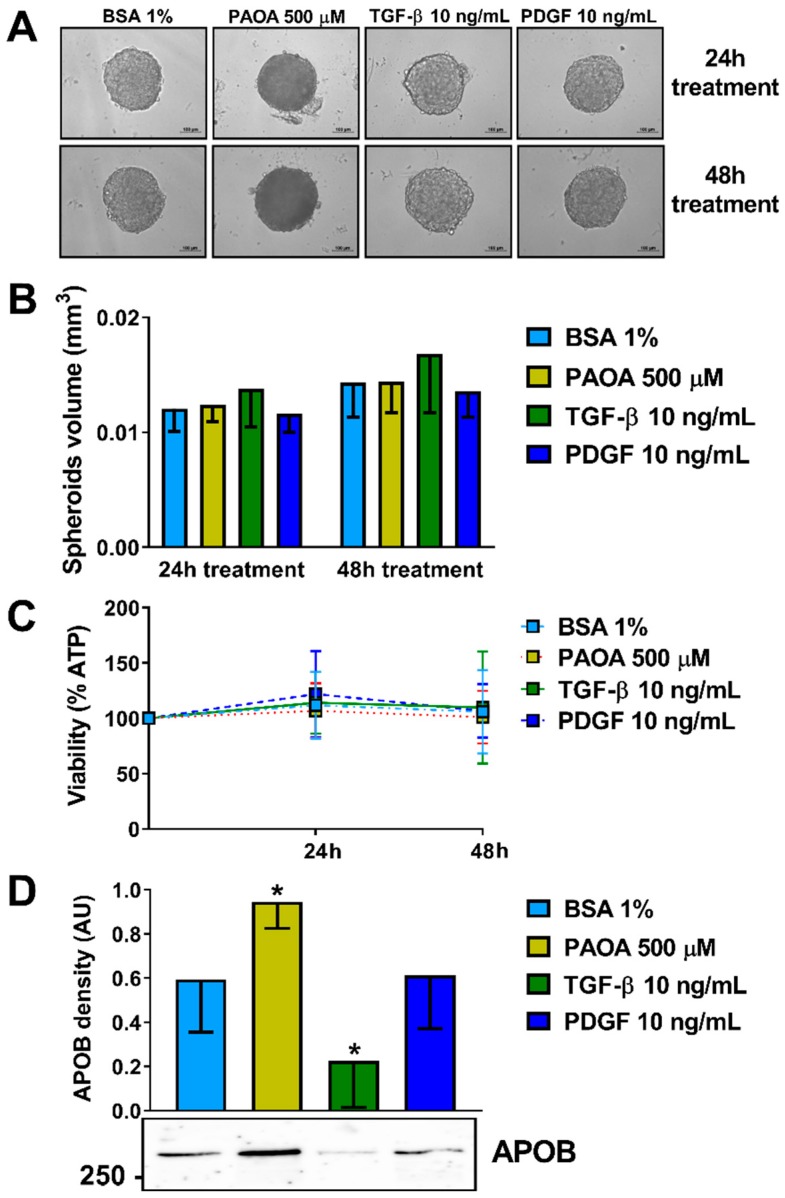
Treatments of organoid with fatty acids do not change organoid volume and viability but enhance APOB secretion. (**A**) 3D spheroids HepG2/LX-2 ratio 24:1 were treated, after 48 h from the seeding with: BSA 1%, a mix of palmitic acid and oleic acid 500 μM (1:2), TGF-β 10 ng/mL or PDGF 10 ng/mL for 24 or 48 h. (**B**) Spheroid volumes were calculated measuring their long and short diameter by ZEN 2.3 Lite software (Zeiss) (*n* = 3). **(C)** Cellular ATP levels normalized to spheroids volumes remained stable throughout 4 days of culture (*n* = 3). (**D**) APOB secretion levels in the media fractions measured by Western blotting were higher in spheroids treated with PAOA while they were lower in spheroids treated with TGF-β (*n* = 7). Bars represent mean ± SD. P-value was calculated by Mann-Whitney non-parametric test, (* *p* < 0.05 vs. BSA 1%). BSA: bovine serum albumin; PAOA: palmitic acid/oleic acid; TGF-β: Transforming growth factor-β; PDGF: Platelet-derived growth factor; APOB: Apolipoprotein B; AU: arbitrary unit.

Initially, we investigated the optimal conditions and timeframe for the induction of steatosis in the multilineage spheroid. We selected the HepG2/LX-2 at a 24:1 ratio, representing the most physiological condition [37]. Spheroids were seeded in regular minimum essential medium (MEM), following spheroid formation at 48 h, they were exposed to four different conditions namely bovine serum albumin (BSA) 1%, a mixture of 500 μM fatty acids palmitic acid and oleic acid (ratio 1:2), Transforming growth factor-β (TGF-β) 10 ng/mL or Platelet-derived growth factor (PDGF) 10 ng/mL for 24 or 48 h (Figure 2A). No difference in spheroids volume was detected (Figure 2B). To test the cell viability of the spheroids after the treatments ATP levels were measured. The ATP levels remained mostly stable in relation to spheroid volume (Figure 2C). Interestingly, fatty acid incubation resulted in higher levels of secreted APOB while incubation with TGF-β in lower levels of APOB in the media (Figure 2D).

**Figure 3:**
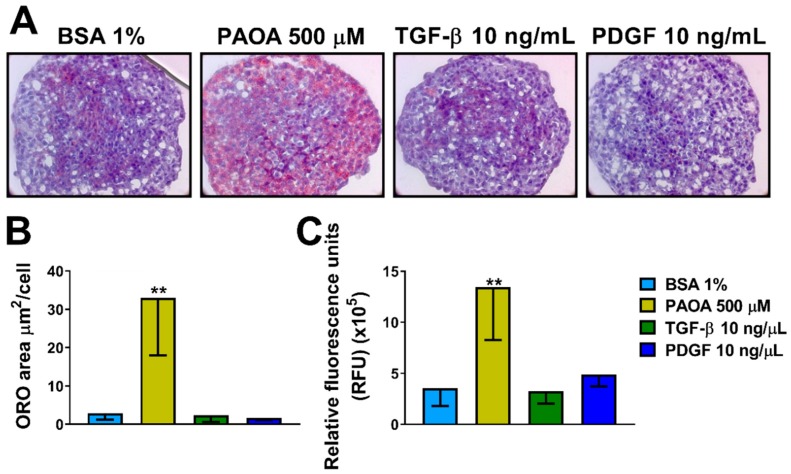
Treatment with fatty acids (PAOA) increases neutral fat content in 3D multilineage spheroids. (**A**) Intracellular neutral lipid content visualized by ORO staining in sections (8 μm) of 3D spheroids HepG2/LX-2 ratio 24:1 treated, after 48 h from the seeding with BSA 1%, a mix of palmitic acid and oleic acid (PAOA) 500 μM (1:2), TGF-β 10 ng/mL or PDGF 10 ng/mL for 48 h. Cell nuclei were stained with hematoxylin. Objective: 40×. (**B**) Quantification of intracellular ORO-stained area quantified by BioPix software (*n* = 5). (**C**) Intracellular lipid content measured by AdipoRed assay kit (Lonza). Bars represent mean ± SD (*n* = 6). P-value was calculated by Mann-Whitney non-parametric test, (** *p* < 0.005 vs. BSA 1%). BSA: bovine serum albumin; PAOA: palmitic acid/oleic acid; TGF-β: Transforming growth factor β; PDGF: Platelet-derived growth factor, ORO: oil red O staining.

To examine if fatty acids, TGF-β or PDGF regulate intracellular neutral fat content, spheroids were incubated with four different conditions including only BSA 1%, a mixture of fatty acids palmitic acid/oleic acid 500 μM (1:2), TGF-β 10 ng/mL or PDGF 10 ng/mL for 48 h (Figure 3A and Figure S1A). The environment with elevated free fatty acids (500 μM) promoted lipid accumulation and an increase in total fat content after 48 h compared to control in medium containing only BSA 1%, measured with Oil Red O (ORO) staining (Figure 3B and Figure S1B). No difference in fat accumulation was detected after treatment with TGF-β or PDGF. To confirm our results, the total fat content of spheroids was measured by AdipoRed assay showing virtually identical results (Figure 3C).

### 2.3. Incubation of Multilineage 3D Spheroids with Fatty Acids Results in Fibrosis

**Figure 4:**
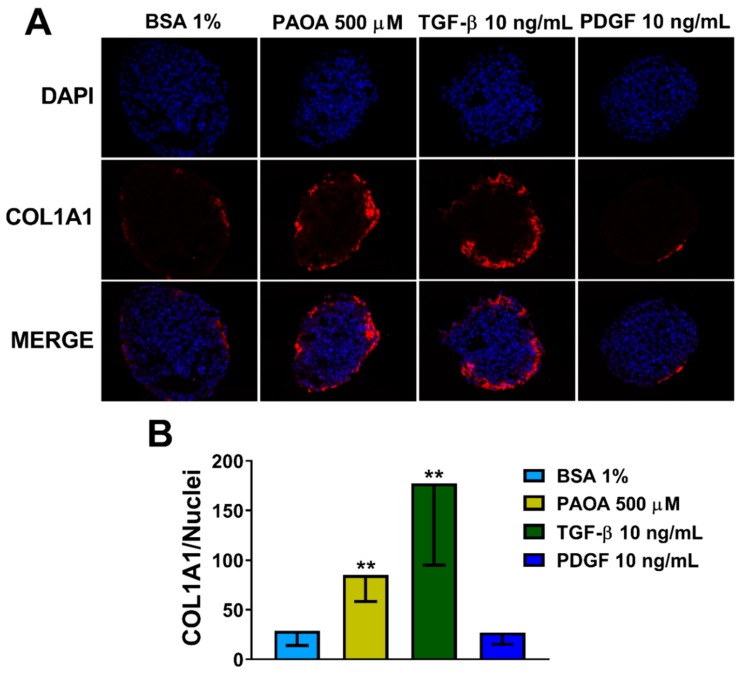
Fatty acid treatment results in an increase in COL1A1 levels in 3D multilineage spheroids. (**A**) Immunofluorescence staining of DAPI (blue), COL1A1 (red) and merged images of 3D spheroids (HepG2/LX-2, 24:1) treated, after 48 h from the seeding, with BSA 1% (negative control), a mix of palmitic acid and oleic acid 500 μM (1:2), TGF-β 10 ng/mL or PDGF 10 ng/mL for 48 h. All the media for treatments were supplemented with BSA 1%. Objective: 40×. (**B**) Quantification of COL1A1 levels by ImageJ normalized to number of nuclei. Bars represent mean ± SD (*n* = 6). P-value was calculated by Mann Whitney non-parametric test, (** *p* < 0.005 vs. BSA 1%). BSA: bovine serum albumin; PAOA: palmitic acid/oleic acid; TGF-β: Transforming growth factor β; PDGF: Platelet-derived growth factor. COL1A1: collagen type I alpha 1.

To show a causal role of steatosis induction on fibrosis development and hepatic stellate cell activation, we measured levels of collagen I (COL1A1) (Figure 4A). Interestingly, we found an increase in COL1A1 production in spheroids treated for 48 h with fatty acids (PAOA) (Figure 4B). Consistently, the levels of COL1A1 were also upregulated with TGF-β, a potent cytokine involved in fibrogenesis. TGF-β is critical for the activation of fibrogenic myofibroblasts, which in response to injury upregulate α-smooth muscle actin (α-SMA) and secrete extracellular matrix proteins, mostly collagen Type I [38,39]. No differences were found after treatment with PDGF. PDGF is a potent factor involved in stimulating HSC proliferation, differentiation, and migration [40].

### 2.4. Prevention of Steatosis in Multilineage 3D Spheroids by Drug Treatment

**Figure 5:**
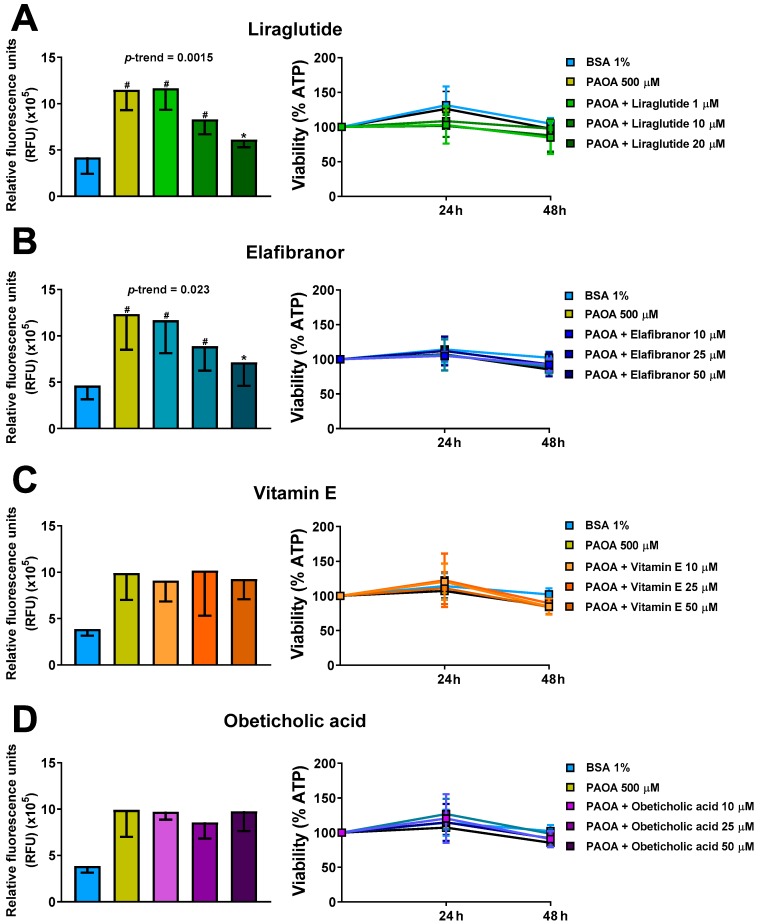
Incubation of 3D multilineage spheroids with liraglutide or elafibranor results in a reduction of intra-spheroid fat content. Prevention of steatosis by co-treatment with (**A**) Liraglutide (1, 10 and 20 μM), (**B**) Elafibranor (10, 25, 50 μM), (**C**) vitamin E (10, 25, 50 μM) or (**D**) obeticholic acid (10, 25, 50 μM) for 48 h in the presence of 500 μM free fatty acids (PAOA) bound to BSA 1%. On the left panels, lipid levels were quantified using AdipoRed biochemical quantification assay. On the right panels cellular ATP levels, normalized to spheroids volumes, remained stable throughout 48 h of treatment (*n* = 3). Bars represent mean ± SD. P-value was calculated by Mann-Whitney non-parametric test, (* *p* < 0.05 vs. PAOA 500 μM; # *p* < 0.005 vs. BSA 1%). BSA: bovine serum albumin; PAOA: palmitic acid/oleic acid.

Having observed induction of intra-spheroid steatosis and accumulation of triglycerides with a consequent increase of COL1A1 levels, we next investigated whether this intra-spheroid steatosis could be prevented by using four compounds for the treatment of NASH, namely liraglutide, elafibranor, vitamin E and obeticholic acid. Liraglutide, elafibranor and obeticholic acid are under evaluation in clinical trials. We found that liraglutide and elafibranor prevented the development of steatosis by at least 50% (*p* < 0.05) compared to untreated controls incubated only with PAOA (Figure 5A,B). Neither vitamin E nor obeticholic acid at physiologically relevant concentrations prevented spheroid fat accumulation (Figure 5C,D). Furthermore, compound exposure did not affect cell viability measured as ATP levels at any of the tested concentrations (Figure 5A–D).

### 2.5. Reduction of COL1A1 Levels by Pharmacological Prevention of Steatosis

**Figure 6:**
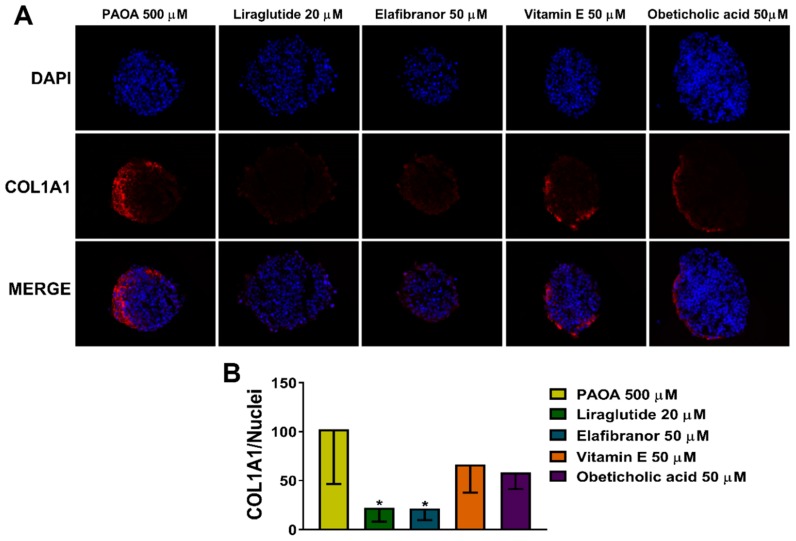
COL1A1 levels decrease in 3D multilineage spheroids treated with liraglutide or elafibranor. (**A**) Immunofluorescence staining of DAPI (blue), COL1A1 (red), and merged images of 3D spheroids (HepG2/LX-2, 24:1) treated, after 48 h from the seeding, with a mix of palmitic acid and oleic acid 500 μM (1:2) bound to BSA 1%, with and without liraglutide 20 μM, elafibranor 50 μM, vitamin E 50 μM or obeticholic acid 50 μM for 48 h. Objective: 40×. (**B**) Quantification of COL1A1 levels by ImageJ normalized to number of nuclei. Bars represent mean ± SD (*n* = 4). P-value was calculated by Mann Whitney test non-parametric test, (* *p* < 0.05 vs. PAOA 500 μM). PAOA: palmitic acid/oleic acid; COL1A1: collagen type I alpha 1.

In order to test whether the COL1A1 levels were regulated by intra-spheroid steatosis, we measured the COL1A1 levels after treatment with drugs as previously described. Interestingly, we found that the COL1A1 levels were decreased after steatosis prevention by liraglutide or elafibranor incubations (Figure 6). On the other hand, we did not observe any reduction in COL1A1 deposition using vitamin E or obeticholic acid that also did not show any effect on prevention of steatosis.

## 3. Discussion

In this study, we implemented an in vitro model of NAFLD with genetic susceptibility, being homozygotes for the *PNPLA3* I148M sequence variant, the strongest genetic determinant of NAFLD. This model consisted of 3D multilineage hepatic spheroids composed by hepatocyte and hepatic stellate cells where we induced fat accumulation and collagen secretion by incubation with free fatty acids. Moreover, we rescued this phenotype by incubating with anti-steatotic and fibrotic drugs, namely liraglutide and elafibranor.

Non-alcoholic fatty liver disease (NAFLD) is an emerging health issue globally affecting a large proportion of the population in many countries [41]. To study NAFLD, in the last decades, several in vitro as well as in vivo models for liver disease and drug discovery have been developed [5,9,10]. However, the commonly used 2D cell culture does not reproduce the complexity of the hepatic tissue while results from in vivo animal models often do not translate to humans, possibly due to differences in physiology [12]. 3D culture systems have recently gained significant attention as a more reliable in vitro system for studying various molecular processes and screening of therapeutic agents [42].

In the present study, we attempted to create a spheroid and to induce intra-spheroid steatosis and fibrosis in it by incubation with free fatty acids. We used multilineage 3D spheroids composed by hepatocytes (HepG2) and hepatic stellate cells (LX-2). HepG2 cells are homozygotes for the *PNPLA3* I148M sequence variant, the strongest genetic determinant of NAFLD [23] expressed at high levels in both hepatocytes and hepatic stellate cells [28,29]. Consistently with Song et al. [17], co-culture of HepG2 with LX-2 enhanced the compactness of spheroids. Indeed, HepG2 cells plated alone were loosely bound to each other during the progression of spheroid assembly due to the lack of ECM-related components such as collagen I, III, and VI [17].

Then, to induce intra-spheroid steatosis, we incubated HepG2 and LX-2 with the most physiological ratio of (24:1), with fatty acids, TGF-β or PDGF. All the treatments did not affect cell viability suggesting that all the doses tested were non-toxic. Furthermore, no differences in spheroid volumes were detected. As expected, incubation with fatty acids induced intra-spheroid fat accumulation. Interestingly, upon incubation with fatty acids, we found increased APOB levels in the media suggesting higher secretion of very-low density lipoprotein (VLDL). This result is consistent with human studies showing that hepatic lipoprotein secretion increases with the increase of liver fat content [43]. On the other hand, upon incubation with TGF-β, we observed a reduction in APOB levels in the media suggesting lower secretion of VLDL. This is consistent with Xu et al. where TGF-β significantly inhibited both *APOM* and *APOB* mRNA expression in HepG2 cells [44].

The intra-spheroid accumulation of fat was confirmed by ORO staining and by biochemical Adipored assay. In 2012, Chavez-Tapia et al. showed that fat-laden hepatocytes have a pivotal role in the initiation of liver fibrosis [45]. Consistently, Giraudi et al. showed that *COL1A1* levels were increased in a 2D co-culture model of hepatocytes and stellate cells after free fatty acids exposure [46]. Here we show for the first time that incubation with fatty acids in 3D spheroids has the same effect. Moreover, we have shown a causal role of liver fat in generating fibrosis by using Mendelian randomization in humans [8].

To investigate the effect of fat accumulation on the acquisition of a fibrogenic phenotype we measured the intra-spheroid type I collagen levels. Type I collagen, the major component of extracellular matrix (ECM) and the most abundant form of collagen in the body, is the most frequently measured collagen component of scar proteins [47]. We found an increase in collagen levels after fatty acids treatment and as expected after treatment with TGF-β [48,49,50] the most potent fibrogenic cytokine [20]. Stellate cells are the major hepatic cells capable of upregulating *COL1A1* mRNA following a fibrogenic stimulus [51]. Increased *COL1A1* mRNA levels were observed in activated cells compared to quiescent hepatic stellate cells in vivo [50,52]. Interestingly, collagen staining was found exclusively in the external margins periphery of the spheroid suggesting that hepatic stellate cells are located primarily in the periphery of the spheroids. This is consistent with the localization of stellate cells in the space of Disse, a small area between the sinusoids and hepatocytes, in human liver [53].

In order to confirm that intra-spheroid steatosis is responsible for fibrosis progression, we tested some compounds in current late-stage clinical development for the treatment of NASH. As of today, we lack approved pharmaceutical treatments of NASH. Nevertheless, there are numerous clinical trials ongoing with drugs that target specific pathomechanisms in NASH [3,41]. The selected compounds are representative of different drug classes and therapeutic concepts and included agonists for the GLP-1 receptor (liraglutide), FXR (obeticholic acid), PPAR-α/δ (elafibranor) [54,55,56] and an antioxidant (vitamin E). Spheroids were incubated with only fatty acids or with fatty acids in combination with different compounds. We found that liraglutide and elafibranor prevented the development of steatosis by at least 50% (*p* < 0.05) compared to untreated control incubated only with fatty acids (PAOA). Neither vitamin E nor obeticholic acid at physiologically relevant concentrations prevented steatosis induction. Afterwards, we measured COL1A1, and we found decreased levels after incubation with liraglutide or elafibranor. On the contrary, no reduction of COL1A1 levels was detected with vitamin E or obeticholic acid. These data suggest that the induction of steatosis has a direct effect on acquisition of fibrogenic phenotype and that the reduction of fibrosis observed in clinical trials with elafibranor is due to a reduction in liver steatosis [56]. A limitation of our study is the use of tumoral and immortalized cells to generate spheroids. However, these cells responded as expected after stimuli with fatty acids and TGF-β indicating a relatively good degree of differentiation. Other advantages of using these cells were the absence of inter-donor variability found in cultures using primary cells, they are easy to use and deliver results in a rapid manner, enabling high-throughput screens in the future.

Tølbøl et al. showed that liraglutide only improved steatosis in a diet specific manner in DIO-NASH mice while elafibranor and obeticholic acid reduced both hepatic steatosis and inflammation and only elafibranor reduced fibrosis severity [57]. We found a reduction in spheroid steatosis and fibrosis after incubation with liraglutide. This is consistent with the presence of GLP-1 receptors in human immortalized hepatocytes, namely HepG2 and Huh7 [58,59,60], and indicates that GLP-1 can have a direct effect on improving NAFLD in the liver independent of its role in body weight reduction. Vitamin E is a potent antioxidant reducing oxidative stress in NAFLD [61]. However, consistently with our results, vitamin E at physiological concentrations did not prevent steatosis induction in human organoids [5].

Studies in murine models showed that the *PNPLA3* I148M sequence variation induces an increase in the lipogenic activity [24] and that downregulation of the protein is protective against NAFLD [25,26,27]. Moreover, carriers of the *PNPLA3* I148M variant have several specific features including a reduction in VLDL secretion [27], retention of retinol in HSCs [28,29,30,31], a specific signature in circulating lipoproteins [32] and a diverse hepatic response to drugs [33,34,35]. All this suggest that *PNPLA3* I148M associated disease represents a separate entity within NAFLD. Therefore, a model homozygous for the mutation may allow the identification of treatment in a framework of precision medicine for this specific group of individuals.

In conclusion, we have established a simple, easy to handle, in vitro model of genetically induced NAFLD consisting of a multilineage 3D spheroid. This tool may be used to understand molecular mechanisms involved in the early stages of fibrogenesis induced by lipid accumulation. Moreover, it may be used to identify new compounds against liver steatosis by high-throughput drug screening followed by more targeted analyses to test the effect on markers of liver fibrosis. Future direction will be to incorporate more cell types into the model to mimic even further the complexity of liver disease and to understand the interplay within the different cell types.

## 4. Materials and Methods

### 4.1. Cell Lines

HepG2 cells were purchased from ATCC (Menassas, VA, USA). After thawing, cells were plated in T-75 flasks and grown in Minimum Essential Medium (MEM) supplemented with 10% Fetal Bovine Serum (FBS), L-glutamine 2 mM, sodium pyruvate 1 mM, non-essential amino acids 1X, penicillin 100 units/mL, and streptomycin 100 μg/mL (HyClone Laboratories, Logan, UT, USA).

When confluent, cells were trypsinated (0.05% trypsin/0.53 mM EDTA) and seeded at a ratio of 1:3. Subsequent passages were performed every 6 days. Immortalized human hepatic stellate cells (LX-2) were purchased from Millipore (Burlington, MA, USA). LX-2 cells were grown in high glucose Dulbecco’s Modified Eagle Medium (DMEM) (HyClone Laboratories) containing 10% fetal bovine serum, penicillin 100 units/mL, and streptomycin 100 μg/mL in T-75 flasks. When confluent, LX-2 cells were sub-cultured like HepG2 cells. The cells were maintained at 37 °C in a humidified atmosphere of 5% CO_2_. HepG2 and LX-2 cells were genotyped for the *PNPLA3* rs738409 and resulted in homozygotes for the 148M allele variant.

### 4.2. D Spheroid Culture

For the generation of the cell spheroids, cells were seeded into 96-well round bottom ultra-low attachment plates (Corning) at 2000 viable cells per well. HepG2, HepG2/LX-2 1:1 and HepG2/LX-2 24:1 spheroids were grown in Minimum Essential Medium (MEM) supplemented as described above. LX-2 spheroids were grown in high glucose Dulbecco’s Modified Eagle Medium (DMEM) supplemented as described above. The plates were incubated for 48 h at 37 °C in a humidified atmosphere of 5% CO_2_. The volume of spheroids was determined using the following formula: 4/3 π r^3^, where “r” was the mean of the long diameter and short diameter of the spheroid divided by two.

### 4.3. Induction of Steatosis

Palmitic acid, oleic acid, PDGF and BSA were purchased from Sigma-Aldrich (St. Louis, MI, USA). TGF-β was purchased from R&D systems. After spheroid aggregation at 48 h after seeding, when the spheroids were compact, HepG2/LX-2 24:1 spheroids were exposed with a mixture of fatty acids palmitic acid and oleic acid 500 μM (1:2) conjugated to BSA, TGF-β 10 ng/mL or PDGF 10 ng/mL for a further 48 h after which spheroids were collected. For all the tested conditions, media were supplemented with bovine serum albumin (BSA) 1%. For conjugation, fatty acids were dried under nitrogen flow and resuspended in medium (1/10 of the desired volume) containing BSA 10% and mixed overnight at 40 °C. The day after, the medium was diluted 1:10 with fresh medium and used to treat the spheroids.

### 4.4. Drug Treatments

Pre-steatotic spheroids were subject to drug treatment. Prevention of steatosis was assessed testing liraglutide (1, 10 and 20 μM), elafibranor (10, 25 and 50 μM), vitamin E (10, 25 and 50 μM) and obeticholic acid (10, 25 and 50 μM). The indicated drugs were purchased from MedChemTronica (MCE) (Sollentuna, Sweden).

### 4.5. Lipid Assay

The AdipoRed Assay Reagent (Lonza, Basel, Switzerland) was used to measure lipid accumulation according to the manufacturer’s instructions. Briefly, spheroids were collected and moved to a new 96-well clear bottom plate with 200 μL of PBS in each well. 20 μL of trypsin was added and incubated at 37 °C for 20 min. Next, 7 μL of AdipoRed reagent was added in each well, mixed by pipetting and incubated for 10 min at room temperature. The fluorescence was analyzed by SpectraMax i3 (Molecular Devices, San Jose, CA, USA) counter with excitation 485 nm and emission 545 nm using SoftMax Pro 6.3 software.

### 4.6. Cell Viability Assay

CellTiter-Glo Luminescent Cell Viability Assay kit (Promega, Madison, WI, USA) was utilized to measure ATP content and thereby cell viability according to the manufacturer’s instructions. Briefly, 50 µL of the reagent was added to each sample well. After disruption of spheroids by pipetting, the plate was incubated at room temperature for 20 min in darkness. Then, the plate was placed in the SpectraMax i3 (Molecular Devices) counter and luminescence was measured using the SoftMax Pro 6.3 software (San Jose, CA, USA).

### 4.7. Immunoblotting

Spheroid media were collected, mixed with Laemmli buffer containing 2-mercaptoethanol and boiled at 95 °C for 5 min. Proteins were size-separated by SDS-PAGE (custom 6% acrylamide gel)—all the reagents were purchased from BioRad (Hercules, CA, USA). Then they were transferred onto nitrocellulose membranes (0.4 A, 2 h). Membranes were incubated for 1 h with mouse anti-APOB (Santa Cruz, sc-13538, Dallas, TE, USA), washed 2 times for 10 min each with 0.2% Tris Buffered Saline containing 0.2% Tween (TBST), incubated 1 h with horseradish peroxidase (HRP)-conjugated secondary antibodies, then washed 3 times for 10 min each with 0.2% TBST. Membranes were incubated for 5 min with chemiluminescent HRP substrate (Millipore Corporation, Billerica, MA, USA), bands were visualized by Chemidoc XRS System (Biorad) and quantified using Image Lab Software (Biorad).

### 4.8. Spheroid and Tissue Imaging

3D spheroids were fixed with 10% *w*/*v* paraformaldehyde (PFA, Sigma-Aldrich) for 2 h, then incubated with 20% *w*/*v* sucrose in Phosphate Buffered Saline (PBS, Lonza) overnight, washed 3 times with PBS, embedded in OCT Cryomount (Histolab, Västra Frölunda, Sweden) and stored at −80 °C. Spheroids were sectioned into 8 µm-thick slices using cryostat (Leica, Wetzlar, Germany). Sections were stored at −80 °C.

### 4.9. Oil Red O (ORO) Staining

The total area of ORO-stained lipid droplets was determined as previously described [60]. Nuclei were stained by hematoxylin or DAPI. Images with hematoxylin-stained nuclei were obtained using Axio Imager M1 (Zeiss) and AxioVision 4.8 Software (Zeiss, Oberkochen, Germany), while images with DAPI-stained nuclei were obtained by Axioplan 2 (Zeiss) using AxioVision 4.8 Software (Zeiss). ORO-stained area was quantified by BioPix iQ 2.1.4 software (BioPix AB, Gothenburg, Sweden) for the images with hematoxylin-stained nuclei, while for the DAPI-stained ones, nuclei were counted, and ORO-stained area was quantified using an in-house macro in ImageJ (v.1.52h, NIH) utilizing a static threshold between all images for determining ORO-positive area within the spheroids.

### 4.10. Immunofluorescence

Sections were incubated with 4% *w*/*v* Bovine Serum Albumin (BSA, Sigma-Aldrich) in PBS for 1 h. Primary antibody anti-COLLAGEN I (Sigma-Aldrich, HPA011795) (1:100) was diluted in 4% *w*/*v* BSA (PBS) and incubated for 1 h at room temperature, followed by 2 washing steps and incubation with fluorescent secondary antibody (Alexa Fluor 594, Invitrogen) (1/1000) for 1 h at room temperature. Nuclei were stained by DAPI (Sigma-Aldrich) (1:8000 in PBS) for 5 min. Finally, cells were mounted with fluorescence mounting medium (Dako). Pictures were obtained using Axioplan 2 (Zeiss) with AxioVision 4.8 Software (Zeiss). Image analysis was performed using an in-house macro in ImageJ (v.1.52h, NIH) where nuclei were counted, the total spheroid area was determined, and a static threshold was applied to all images for each of the fluorescent channels to determine positively stained area.

### 4.11. Statistical Analysis

Data from in vitro experiments were analyzed using the Mann-Whitney non-parametric test. *P*-values of <0.05 were considered significant and indicated as * in figures, or if <0.005 as **. Bar graphs in figures show mean ± SD of at least three experiments unless specified otherwise.

## Supplementary Materials

Supplementary Materials can be found at https://www.mdpi.com/1422-0067/20/7/1629/s1.
