# Supplementary material for: Human Multilineage 3D Spheroids as a Model of Liver Steatosis and Fibrosis
Source: Int J Mol Sci. 2019 Apr 2;20(7):1629. doi: 10.3390/ijms20071629 (PMC6480107; doi:10.3390/ijms20071629)
Supplement: Supplementary file 1 [file ijms-20-01629-s001.pdf]

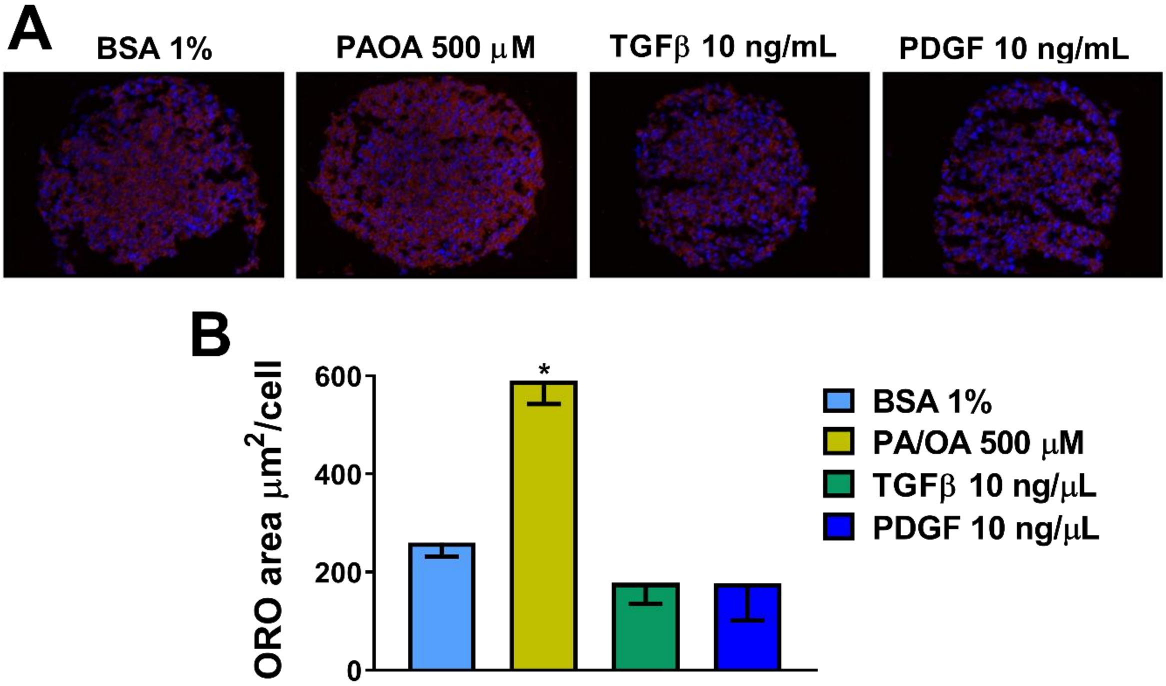

Supplementary Figure 1: Treatment with fatty acids (PAOA) increases neutral fat content in 3D multilineage spheroids. (A) Intracellular neutral lipid content visualized by ORO staining in sections (8 μm) of 3D spheroids HepG2/LX-2 ratio 24:1 treated, after 48 hours from the seeding with BSA 1%, a mix of palmitic acid and oleic acid (PAOA) 500 μM (1:2), TGF-β 10 ng/mL or PDGF 10 ng/mL for 48 hours. Cell nuclei were stained with DAPI. **(B)** Quantification of intracellular ORO-stained area quantified by ImageJ software (n=4). P-value was calculated by Mann-Whitney non-parametric test, (* $p<0.05$ vs BSA 1%). BSA: bovine serum albumin; PAOA: palmitic acid/oleic acid; TGF-β: Transforming growth factor β; PDGF: Platelet-derived growth factor; ORO: oil red O staining.
